# Supplementary material for: Wetland vegetation composition and ecology of Lake Abaya in southern Ethiopia
Source: PLoS One. 2024 Apr 10;19(4):e0301795. doi: 10.1371/journal.pone.0301795 (PMC11006174; doi:10.1371/journal.pone.0301795)
Supplement: Attachment — Submitted filename: PONE-D-23-20697_Review.pdf [file pone.0301795.s002.pdf]

PLOS ONE

Wetland Vegetation Composition and Ecology of Lake Abaya in Southern Ethiopia

--Manuscript Draft--

**Manuscript Number:**	PONE-D-23-20697
**Article Type:**	Research Article
**Full Title:**	Wetland Vegetation Composition and Ecology of Lake Abaya in Southern Ethiopia
**Short Title:**	Wetland Vegetation Composition and Ecology
**Corresponding Author:**	Dikaso Unbushe Gojamme, Ph.D.<br>WSU: Wolaita Sodo University<br>Wolaita Sodo, ETHIOPIA
**Keywords:**	Wetland; Floristic composition; Plant community; Lake Abaya; Vegetation
**Abstract:**	Wetland vegetation and ecology of Lake Abaya in the southern Ethiopia was studied to determine floristic composition, plant community type and vegetation ecology. A total of 102 plots were laid along transects that were set up preferentially across areas where there were rapid changes in vegetation or marked environmental gradients to collect data on estimate of percentage aerial cover of plant species and environmental variables. Vegetation data was analyzed by agglomerative hierarchical cluster analysis using similarity ratio as a resemblance index and Ward's linkage method. Multivariate data analysis was performed using appropriate packages in R version 2.14.0. Canonical Correspondence Analysis (CCA) was used to explore the relationship between the species composition and environmental variables. The environmental data included in the CCA were determined using stepwise backward and forward selection of variables by ANOVA test. Statistical measurement regarding species diversity, richness and evenness of the plant community types was carried out by using Shannon-Wiener (1949) diversity indices. A total of 92 plant species belonging to 66 genera and 34 families were identified. Families Poaceae, Asteraceae, Fabaceae, Cyperaceae, Solanaceae, Euphorbiaceae and Amaranthaceae account for about 56.99% of the total proportion. Based on the cluster analysis, five plant community types were identified. The most important factors influencing the plant species composition and pattern of wetland plant communities were water drainage, water depth, land use, slope, altitude, and hydrogeomorphology. Therefore, these factors should be considered in future management and protection under the circumstance of climate change and human activities.
**Order of Authors:**	Dikaso Unbushe Gojamme, Ph.D.
**Additional Information:**	
**Question**	**Response**
**Financial Disclosure**<br><br>Enter a financial disclosure statement that describes the sources of funding for the work included in this submission. Review the submission guidelines for detailed requirements. View published research articles from *PLOS ONE* for specific examples.<br><br>This statement is required for submission and **will appear in the published article** if the submission is accepted. Please make sure it is accurate.	NO - Include this sentence at the end of your statement: The funders had no role in study design, data collection and analysis, decision to publish, or preparation of the manuscript.

Powered by Editorial Manager® and ProduXion Manager® from Aries Systems Corporation

**Unfunded studies**<br>Enter: *The author(s) received no specific funding for this work.*<br><br>**Funded studies**<br>Enter a statement with the following details:<br>• Initials of the authors who received each award<br>• Grant numbers awarded to each author<br>• The full name of each funder<br>• URL of each funder website<br>• Did the sponsors or funders play any role in the study design, data collection and analysis, decision to publish, or preparation of the manuscript?<br>• **NO** - Include this sentence at the end of your statement: *The funders had no role in study design, data collection and analysis, decision to publish, or preparation of the manuscript.*<br>• **YES** - Specify the role(s) played.<br><br>* typeset	
**Competing Interests**<br><br>Use the instructions below to enter a competing interest statement for this submission. On behalf of all authors, disclose any competing interests that could be perceived to bias this work—acknowledging all financial support and any other relevant financial or non-financial competing interests.<br><br>This statement is required for submission and **will appear in the published article** if the submission is accepted. Please make sure it is accurate and that any funding sources listed in your Funding Information later in the submission form are also declared in your Financial Disclosure statement.<br><br>View published research articles from *PLOS ONE* for specific examples.	The authors have declared that no competing interests exist.

**NO authors have competing interests**<br><br>Enter: *The authors have declared that no competing interests exist.*<br><br>**Authors with competing interests**<br><br>Enter competing interest details beginning with this statement:<br><br>*I have read the journal's policy and the authors of this manuscript have the following competing interests: [insert competing interests here]*<br><br>* typeset	
**Ethics Statement**<br><br>Enter an ethics statement for this submission. This statement is required if the study involved:<br><br>• Human participants<br>• Human specimens or tissue<br>• Vertebrate animals or cephalopods<br>• Vertebrate embryos or tissues<br>• Field research<br><br>Write "N/A" if the submission does not require an ethics statement.<br><br>General guidance is provided below. Consult the submission guidelines for detailed instructions. **Make sure that all information entered here is included in the Methods section of the manuscript.**	N/A

Powered by Editorial Manager® and ProduXion Manager® from Aries Systems Corporation

**Format for specific study types**<br><br>**Human Subject Research (involving human participants and/or tissue)**<br>• Give the name of the institutional review board or ethics committee that approved the study<br>• Include the approval number and/or a statement indicating approval of this research<br>• Indicate the form of consent obtained (written/oral) or the reason that consent was not obtained (e.g. the data were analyzed anonymously)<br><br>**Animal Research (involving vertebrate animals, embryos or tissues)**<br>• Provide the name of the Institutional Animal Care and Use Committee (IACUC) or other relevant ethics board that reviewed the study protocol, and indicate whether they approved this research or granted a formal waiver of ethical approval<br>• Include an approval number if one was obtained<br>• If the study involved *non-human primates*, add *additional details* about animal welfare and steps taken to ameliorate suffering<br>• If anesthesia, euthanasia, or any kind of animal sacrifice is part of the study, include briefly which substances and/or methods were applied<br><br>**Field Research**<br><br>Include the following details if this study involves the collection of plant, animal, or other materials from a natural setting:<br>• Field permit number<br>• Name of the institution or relevant body that granted permission	
**Data Availability**<br><br>Authors are required to make all data underlying the findings described fully available, without restriction, and from the time of publication. PLOS allows rare exceptions to address legal and ethical concerns. See the PLOS Data Policy and FAQ for detailed information.	No - some restrictions will apply

Powered by Editorial Manager® and ProduXion Manager® from Aries Systems Corporation

A Data Availability Statement describing where the data can be found is required at submission. Your answers to this question constitute the Data Availability Statement and **will be published in the article**, if accepted.<br><br>**Important:** Stating 'data available on request from the author' is not sufficient. If your data are only available upon request, select 'No' for the first question and explain your exceptional situation in the text box.<br><br>Do the authors confirm that all data underlying the findings described in their manuscript are fully available without restriction?	
**Describe where the data may be found in full sentences. If you are copying our sample text, replace any instances of XXX with the appropriate details.**<br><br>• If the data are **held or will be held in a public repository**, include URLs, accession numbers or DOIs. If this information will only be available after acceptance, indicate this by ticking the box below. For example: *All XXX files are available from the XXX database (accession number(s) XXX, XXX.*).<br>• If the data are all contained **within the manuscript and/or Supporting Information files**, enter the following: *All relevant data are within the manuscript and its Supporting Information files.*<br>• If neither of these applies but you are able to provide **details of access elsewhere**, with or without limitations, please do so. For example:<br><br>*Data cannot be shared publicly because of [XXX]. Data are available from the XXX Institutional Data Access / Ethics Committee (contact via XXX) for researchers who meet the criteria for access to confidential data.*<br><br>*The data underlying the results presented in the study are available from (include the name of the third party*	All relevant data are within the manuscript

and contact information or URL).<br>• This text is appropriate if the data are owned by a third party and authors do not have permission to share the data.<br><br>* typeset	
Additional data availability information:	

Powered by Editorial Manager® and ProduXion Manager® from Aries Systems Corporation

Manuscript

Click here to access/download;Manuscript;Wetland Vegetation and Ecology of lake Abaya.docx

Wetland Vegetation Composition and Ecology of Lake Abaya in Southern Ethiopia

Dikaso Unbushe Gojamme

Wolaita Sodo University, College of Natural Sciences, P.O.Box. 138, Wolaita Sodo, Ethiopia

dikaso.unbushe1@wsu.edu.et; unbusheg@gmail.com; https://orcid.org/0000-0002-0526-3147

Telephone: +251911752316

Abstract

Wetland vegetation and ecology of Lake Abaya in the southern Ethiopia was studied to determine floristic composition, plant community type and vegetation ecology. A total of 102 plots were laid along transects that were set up preferentially across areas where there were rapid changes in vegetation or marked environmental gradients to collect data on estimate of percentage aerial cover of plant species and environmental variables. Vegetation data was analyzed by agglomerative hierarchical cluster analysis using similarity ratio as a resemblance index and Ward's linkage method. Multivariate data analysis was performed using appropriate packages in R version 2.14.0. Canonical Correspondence Analysis (CCA) was used to explore the relationship between the species composition and environmental variables. The environmental data included in the CCA were determined using stepwise backward and forward selection of variables by ANOVA test. Statistical measurement regarding species diversity, richness and evenness of the plant community types was carried out by using Shannon-Wiener (1949) diversity indices. A total of 92 plant species belonging to 66 genera and 34 families were identified. Families Poaceae, Asteraceae, Fabaceae, Cyperaceae, Solanaceae, Euphorbiaceae and Amaranthaceae account for about 56.99% of the total proportion. Based on the cluster analysis, five plant community types were identified. The most important factors influencing the plant species composition and pattern of wetland plant communities were water drainage, water depth, land use, slope, altitude, and hydrogeomorphology. Therefore, these factors should be considered in future management and protection under the circumstance of climate change and human activities.

Key words: *Wetland, Floristic composition, Plant community, Lake Abaya, Vegetation*

1. Introduction

Irrespective of the size and spatial distribution, wetlands provide a wide range of important values and functional attributes. Wetlands are among the most important productive ecosystems on earth because of the complex interactions between biotic (fauna, flora, microbes and unicellular organisms) and abiotic (soil, water and topography) components of wetland ecosystems (Mitsch and Gosselink 2002; Costanza *et al.* 1997). Wetland ecosystem is considered to be more productive than the adjacent area because of the periodic inflow of nutrients. Their productivity is comparable to rainforests and coral reefs (Costanza *et al.* 1997). They constitute a resource of great economic, cultural, scientific and recreational values. Schuyt (2004) estimated that freshwater wetlands hold more than 40% of the entire world's plant species and 12% of all animal species. Finlayson and Moser (1991) estimated that more than 40% of fishes (of the 20,000 species in the world) live in fresh water wetlands. Individual wetlands can be important in supporting high numbers of endemic species; for example, Schuyt (2004) reported that Lake Tanganyika in Central Africa supports 632 endemic fishes and other animal species.

Wetlands produce an ecological equilibrium in the environment by maintaining the integrity of life support systems for sustainable socio-economic development. Wetlands are also important sources of water for agricultural, industrial and domestic uses. They play important roles in ecological functions in their natural states, which contribute to the wellbeing of human society.

Despite the will in understanding of wetlands and their ecological importance, degradation of wetlands continues, mainly due to anthropogenic activities. About half the global wetland area has been lost as a result of human activities (OECD 1996). In tropical and subtropical areas conversions of wetlands to alternative land uses have accelerated wetland loss and agriculture is considered the principal cause for wetland loss. Wetlands were drained to control disease and for agricultural activities (Maltby 1986; Lean *et al.* 1990; Mitsch and Gossenlink 1993; Wetzel 1975). Other important reasons for their vulnerability are the fact that they are dynamic systems undergoing continual change (Barbier *et al*. 1996) and are the fact that they are often open-access resources with limited control over how they are used and what is harvested from them (Turner *et al*. 2000).

In Ethiopia, wetlands are distributed across all agro-ecological Zones from high altitude of 4000 m above sea level to 125 m below sea level (Afework Hailu *et al*. 2000). The different geological formation, ecological diversity and climatic conditions have endowed Ethiopia with all types of wetlands except coastal, marine-related wetlands and extensive swamp-forest complexes (EPA 2003).

Although wetlands in Ethiopia cover only a small area, they have immense economic, social, and environmental benefits. Wetlands are the main sources of valuable water resources in Ethiopia, where water resources are unevenly distributed and only a quarter of its population has access to safe water. In many parts of Illubabor and Wollega, many perennial and annual springs are associated with the existence of wetlands. Throughout the country, wetlands are important sites for livestock and wildlife grazing especially during the dry period. Floodplains of Borkena, Dabus and Fogera are vital sources of fodder, particularly during dry season, to both domestic and wild animals.

Wetlands are also vital sources of food, fuelwood, and raw materials for making household furniture. Growing number of people in our country, in both rural and urban areas depend on wetland resources for their survival. Poor rural households, particularly women depend on wetlands for additional income to their families. In the ethnic group known as the ‘Agnuak’ in the Gambella lowlands along banks of the Baro and Gello Rivers, women are heavily involved with fishing activities (Afework Hailu *et al*. 2000). Many peasant farmers in the western parts of the country make their living from wetlands. Sedges are one of the important wetland resources that local communities use in different parts of the country. For example, in western Oromia, sedges have great importance for thatching of houses. More than three fourth of the local households in Illubabor Zone (Afework Hailu *et al*. 2000), use sedges for roofing their houses.

Wetlands associated with lake Abaya provide range of ecological and economic importance to wildlife and people in the surrounding area. The wetland vegetation is an important nesting and feeding areas for hundreds of wetland birds and hippopotamus and protective shelter for spawning areas for crocodiles. Wetlands of Rift Valley Lakes are sites for tourist attraction.

Despite the global importance, wetlands in Ethiopia are still facing many problems. The major threat comes from the over harvesting of wetland resources, the expansion of human settlements in the main Ethiopian Rift Valley Lakes (Ziway-Shala, Hawassa, Abaya-Chamo and Chew Bahir basins), the construction of dams in Koka and Melka-Wakana, drainage for agriculture in southwest Ethiopia especially in Jimma and Wollega Zones (Afework Hailu *et al*. 2000).Wetland resources such as water, fishes and vegetation are subjected to over exploitation. Excessive exploitation of the resources from wetlands can lead to a direct collapse of the wetland and its resources. Disappearance of Lake Haramaya wetlands through excessive use of water for irrigation can be evidence to demonstrate overuse of a resource from wetlands.

Threats to wetlands also originate from the catchments since wetlands are closely interacting with the catchment. Wetlands in the lowland areas are threatened by encroachment due to shortage of cultivation and grazing areas that results from population pressure in the highlands. Overgrazing by livestock and wildlife leads to loss of biodiversity

and compaction of the wetland soil during wet periods which can affect infiltration capacity of the wetland soil. This can in turn affect biodiversity of the wetland. In other instances, catchments are the source of agricultural discharges and will result in increased nutrient load to the wetlands (eutrophication) which leads to the colonization (homogenization) of the habitat by single species which are usually invasive (either exotic or indigenous species). Invasive species are a major threat to global biodiversity and an important cause of biotic homogenization of ecosystems (Enserink 1999). Invasive species, either exotic or indigenous, are also threats to wetlands of Ethiopia. Examples of invasive species threatening Ethiopia's wetlands include *Prosopis juliflora* in Awash River basin, *Mimosa pigra* in the Baro-Akobo basin, and water hyacinth, *Eichhornia crassipes*, in Koka reservoirs (Mitiku Tikssa *et al*. 2010; Bayafers Tamene *et al*. 2000). Increased accumulation and sedimentation will ultimately accelerate the rate of conversion of the wetland system to a terrestrial one (Wetzel 1975). The low-level awareness of communities regarding the benefits of wetlands, capacity limitations such as lack of skilled manpower, scarcity of wetland focused and coordinated institutions, lack of technical and financial support for wetlands conservation also accelerate loss of wetlands.

Although wetlands are among the most productive ecosystems on earth and protection of threatened natural wetlands and preservation of its biodiversity has received increasing attention globally, wetlands and their resources in Ethiopia are still facing many problems. High population densities within the catchments of the Ethiopian Rift Valley Lakes and in the highlands have been associated with a series of deleterious trends, in particular those arising from the clearance of vegetation for agriculture and overgrazing. Some of the key challenges to the wetlands of lake Chamo are intensive use of land in the buffer zones for crop production, land loss due to deforestation in the watershed, and eutrophication by nutrients from agricultural fields (Seleshi Bekele 2001). Overgrazing of lakeshore vegetation by livestock and overharvesting particular plant species such as *Aeschynomene elaphroxylon* for the construction of traditional boat, clearance of lakeshore vegetation and trampling due to fishing activities and deforestation in the watershed are also the main threats to the wetlands. The western parts of the two lakes are extensively used for big state farms, which were recently given for private investors.

Despite their importance to maintenance of biodiversity, wetlands and their resources (vegetation in particular) in Ethiopia have been barely investigated and their previous documentation was extremely limited. The vegetation ecology, species composition and diversity of wetlands in Ethiopia have not yet been in the detail it deserved. This is typically reflected in wetlands associated with Lake Chamo in south Ethiopia where research in vegetation ecology, species composition and diversity have not yet been conducted.

Thus, this study was conducted with the purpose of determining the floristic composition, plant species diversity and richness, plant community types, trends in relationship between plant communities and ecological gradients and to recommend some corrective measures of sustainable management. It is also hoped that this research can provide baseline information for further studies and the issues raised and recommendations given in the study areas will apply to the other wetlands in Ethiopia and neighboring countries in the Horn of Africa.

2. Methods and materials

2.1 Study area

Lake Abaya is part of the Ethiopian Rift Valley Lakes found in southern Ethiopia (Figure 1). Lake Abaya is the largest lake in the Ethiopian Rift, located between 5°3'19"N and 6°45'11"N latitude and 37°18'55"E and 38°7'55"E longitude (EMA, 1988). It has a maximum length of 79.2 km, maximum width of 27.1 km with a surface area of 2600 km2 (Seleshi Bekele, 2001). It has a maximum depth of 24.5 m and is located at an average altitude of 1,235 m.a.s.l. (Seleshi Bekele, 2001).

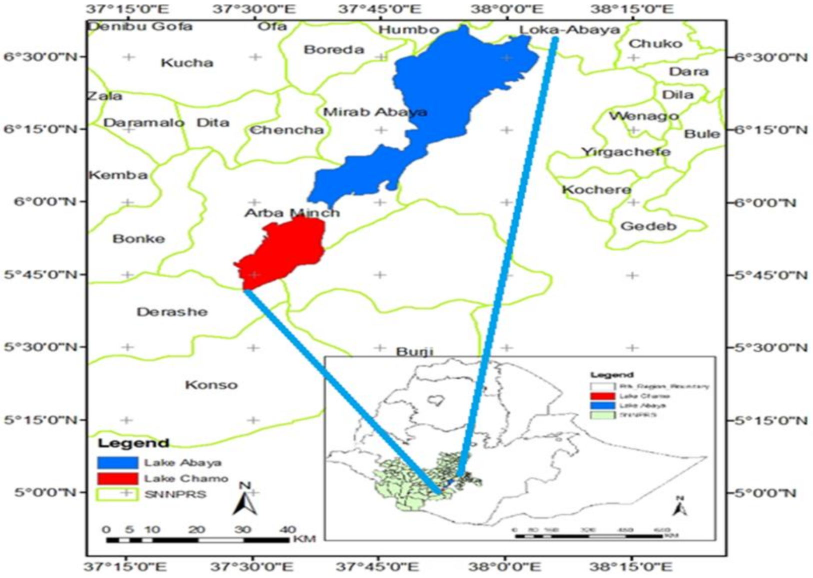

Figure 1: Map showing location of Lake Abaya.

The Abaya-Chamo lakes basin experiences bimodal rainfall pattern (Figure 2). Lake Abaya-Chamo basin is covered by different vegetation types and experiences different land use practices. The land use of Lake Abaya and Chamo wetlands, especially western side of the two lakes, has been changed rapidly due to extensive deforestation as a result of increased number of populations in the area (Seleshi Bekele 2001). The extensive deforestation resulted in the replacement of vegetation cover by cultivated lands. Farming activities like livestock rearing and crop production are the main land use practices in the catchment surrounding the lakes.

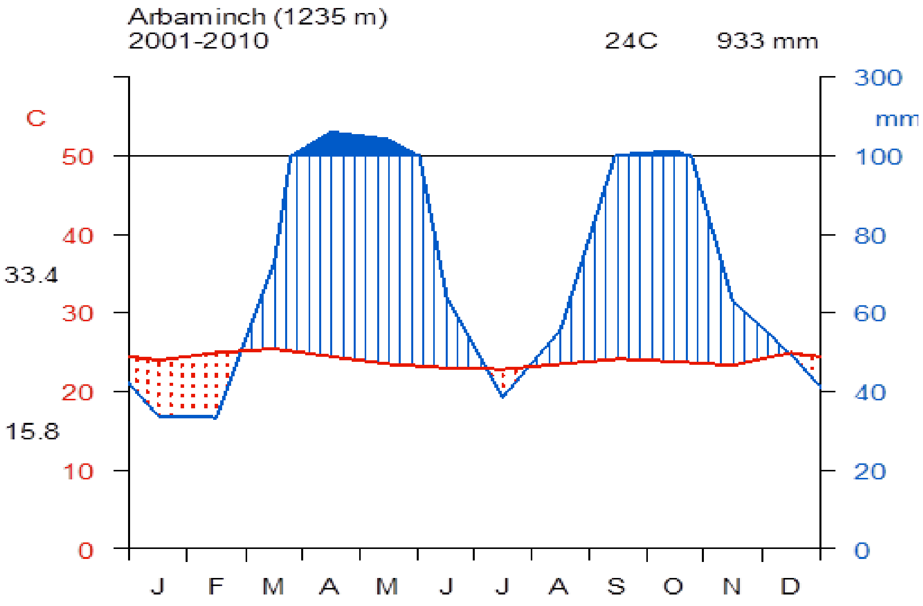

Figure 2: Climate diagram for the study area.

2.2. Data Collection

2.2.1. Vegetation Data Collection

Representative and relatively homogeneous vegetation units of sampling sites were purposively selected on the basis of physiography and physiognomy. Transects were set up preferentially across area where there were rapid changes in vegetation or marked environmental gradients following Muller-Dombois and Ellenberg (1974). Transects were laid out to represent hydrological variations and habitat heterogeneity. Transects were started from the edge of the open water and extended to the ecotone along the edge on both sides of the wetland.

Mueller-Dombois and Ellenberg (1974) and Kent and Coker (1992) suggested quadrat size for meadow and fen vegetation type is 4 m x 4 m (16 m^2) and a shrubby heath, tall herbs with grassland vegetation type are 10 m x 10 m (100 m^2). Thus, because of differences in vegetation zonation pattern and continuity of substrates, vegetation data were collected at each site using a plot size that meets the minimum area requirements for meadow, shrubby heaths, tall herbs with grassland vegetation and shrubby swamps (100m^2).

Sample plots were placed along transects in each of the purposively (preferentially) selected habitat units or strata based on moisture gradient and dominant vegetation types. Location of the plots was chosen visually and purposively to ensure that it gives a representative view of the plant species and abundance in that unit. The location of each plot was recorded using a GPS. Within each sample plot in all study areas, all plant species were recorded and the percentage aerial cover of each species was estimated. This was later converted to 1-9 scale following the Braun-Blanquette method modified by Van der Maarel (1979)

Cover was estimated as the percentage of sampling area covered by the vertical projection of individuals of each species present (Mueller-Dombois & Ellenberg 1974). Plant species occurring outside the plots were also collected and identified to produce a comprehensive list of the plant species diversity of the study area. Voucher specimens were collected, coded, pressed and dried for subsequent identification and verification at the National Herbarium (ETH), Addis Ababa University, using Flora of Ethiopia and Eritrea and those of other neighboring countries.

2.2.2. Environmental data collection

The environmental variables recorded in each plot include: drainage, hydrogeomorphology, disturbance, slope and elevation. At each site or sample plot, estimate of disturbance intensity was recorded based on physical evidence of the site characteristics (e.g., soil irregularities, burning, defoliation and dung present). Plot level hydrogeomorphologic features were rated based on physical evidence of the site or sample plot (e.g., position of the land scape and additional water source) whereas drainage was recorded based on water holding capacity of the soil or level of saturation. Depth to water table was measured in each plot with a labeled PVC pipe. Environmental data on topographic parameters such as altitude and coordinates for each plot were determined with GPS.

2.3. Data Analysis

Multivariate data analysis methods were used to analyze the vegetation and environmental data. Statistical analysis was performed in the R version 2.14 statistical computing program (R Development Core Team 2009) using packages for classification and ordination. Both ordination and classification techniques were employed to study the ecology of wetland vegetation.

Vegetation data were analyzed using agglomerative hierarchical cluster analysis (Mueller-Dombois and Ellenberg 1974) using similarity ratio as a resemblance index and Ward's linkage method to identify vegetation assemblages. Distinct clusters were identified at appropriate hierarchical levels and the quadrats of the data set were then arranged using the sequence of the quadrats in the dendrogram produced. The mean cover value of each species in each

cluster identified was calculated and a synoptic table was produced. The species with highest mean cover value was used to determine dominance and sub-dominance of species in cluster groups. Based on the cluster analysis output and the resulting synoptic table and ecological evaluation in the field, the number of community types (clusters) was determined at appropriate dissimilarity levels (height of the dendrogram). The plant community types were named after one, two or three dominant species, using the highest mean cover values of plant species which occur in each group. Dominant species are those that are most conspicuous in the community and are high in one or more of the importance values (Whittaker 1975), mean cover value in this case.

The identified plant community types were tested for the null hypothesis of no significant difference between the groups using the Multi-response Permutation Procedures (MRPP) (ter Braak and Šmilauer 2002; Ludwig and Reynolds, 1988). A significance level of 0.05 was used to determine if the compositional differences between groups were statistically significant. The Plant community types were examined using non-metric multidimensional scaling (NMDS) ordination. NMDS was chosen because the tests can produce robust visualizations of data despite numerous zero-values and highly variable data with lack of normality (McCune and Grace 2002). Using the synoptic table and the habitat information gathered during the sampling period, the different plant communities were described.

Canonical Correspondence Analysis (CCA) (ter Braak 1987, 1988 and 1990; Hill 1979) was used for revealing patterns in the species composition data and relating the patterns to measured environmental variables. CCA technique assumes a unimodal distribution of species in relation to environmental variables (McCune and Grace, 2002; McCune and Mefford 1997). Species cover abundance data for each plot together with the corresponding plot versus environmental variables data matrix were subjected to Canonical Correspondence Analysis (CCA) to reveal the relations between the species composition and environmental variables.

The environmental data included in the CCA were determined using stepwise backward and forward selection of variables by ANOVA test. The CCA generated biplot scores (i.e., correlations between environmental variables and ordination axes) were used to infer the relative importance of each environmental variable for prediction of species composition and distribution (ter Braak 1995).

2.3.2. Diversity Analysis

Statistical measurement regarding species diversity, richness and evenness of the plant community types was carried out by using Shannon-Wiener (1949) diversity index. The Shannon diversity index (H') was computed using the mean cover values of the species in the plant community types as the input matrix (synopsis output). The Shannon diversity index is calculated by using the formula:

Shannon diversity index $H' = -\sum_{i=1}^{s} Pi \ln Pi$ Where s= the number of species; P_i = the proportion of the individuals of the ith species or the abundance of the ith species expressed as a proportion of total cover; Ln= log $base_n$ (natural logarithm). Equitability which determines the relative evenness of the species within the plant community was also calculated. Equitability (Shannon evenness, J) is calculated by the following formula:

Evenness index $J = \frac{H'}{H'max} = \frac{-\sum_{i=1}^{s} Pi \ln Pi}{LnS}$ Where, J = the equitability; LnS= log $base_n$; S = the number of species

Floristic similarities with regard to species composition among and between study sites were calculated by employing Sorenson’s similarity coefficient (Kent and Coker 1992) by using the equation: SC = 2a/ (2a + b + c) Where SC = Sorenson’s similarity coefficient; a = number of species common to both categories; b = number of species present in the first category and absent in the second and c = number of species present in the second category and absent in the firs

3. Results

3.1. Floristic Composition

A total of ninety two plant species belonging to 66 genera and 34 families were recorded from the wetlands of Lake Abaya. Families Poaceae, Asteraceae, Fabaceae, Cyperaceae, Solanaceae, Euphorbiaceae and Amaranthaceae account for about 56.99% of the total proportion (Figure 3). Poaceae was represented by 14 (15.05%) species belonging to 11 genera; Cyperaceae by 13 species (13.98%) belonging to 2 genera; Fabaceae was represented by 7 species belonging to 4 genera while Asteraceae was represented by 6 species belonging to 6 genera. Euphorbiaceae was represented by 5 species belonging to 4 genera; Solanaceae was represented by 4 species belonging to 4 genera and Amaranthaceae was represented by 4 species belonging to 2 genera.

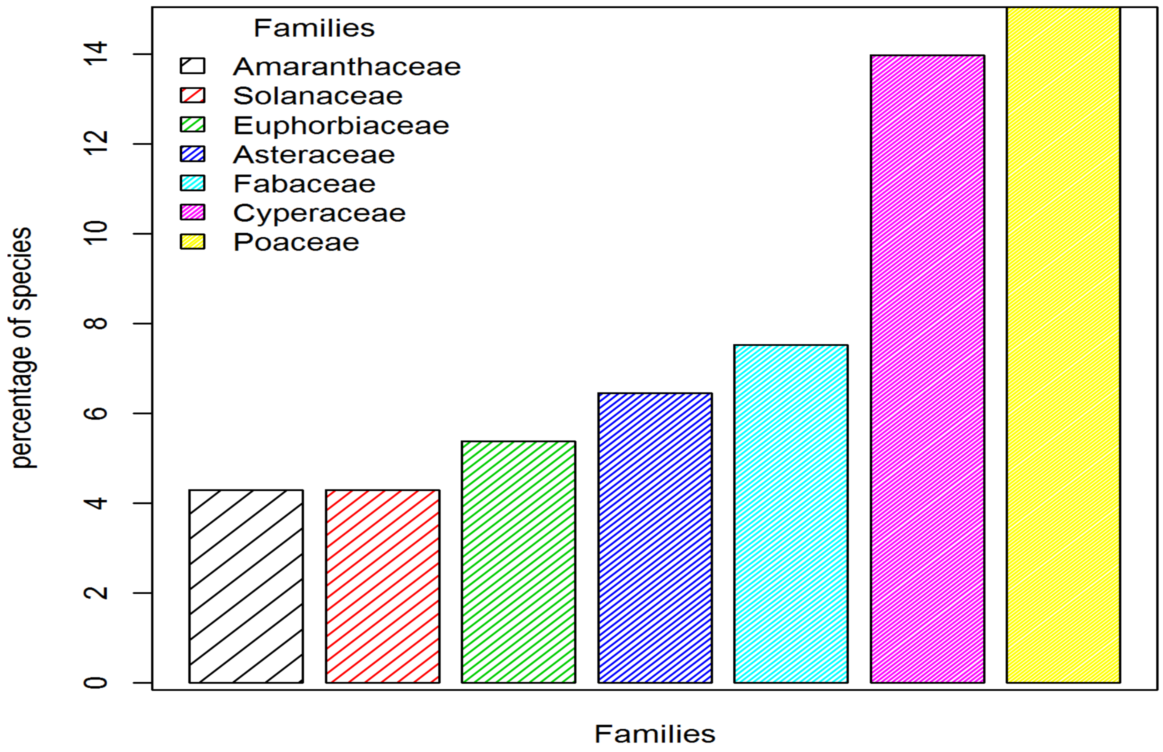

Figure 3: Families with the greatest representation in Lake Abaya wetland

3.1.1. Description of Plant Community Types of Lake Abaya Wetland

Based on the cluster analysis output and ecological evaluation in the field, five community types were identified between 2.0 to 2.5 heights (0.60 - 0.75 dissimilarity levels) of the dendrogram (Figure 4). The plant community types were named by the dominant species, which occur in each group, using the highest synoptic values of plant

species (Table 1). Communities with their dominant and sub-dominant species, the number of relevés they contained and diversity index are given in Table 2.

The most common herbaceous species based on mean cover values (Table 1) included *Cynodon aethiopicus, Cyperus articulatus, Cyperus latifolius, Ipomoea aquatica, Leersia hexandra, Eichhornia crassipes, Pistia stratiotes, Cyperus laevigatus, Typha angustifolia* and *Leptochloa fusca.* Species such as *Aeschynomene elaphroxylon, Cynodon aethiopicus, C. articulatus, C. laevigatus, C. latifolius, Cyperus subumbellatus, Eichhornia crassipes, Leersia hexandra, Leptochloa fusca, Pistia stratiotes,* and *Typha angustifolia* were widely and frequently distributed. Each of these species occurred in more than 10% of the quadrats.

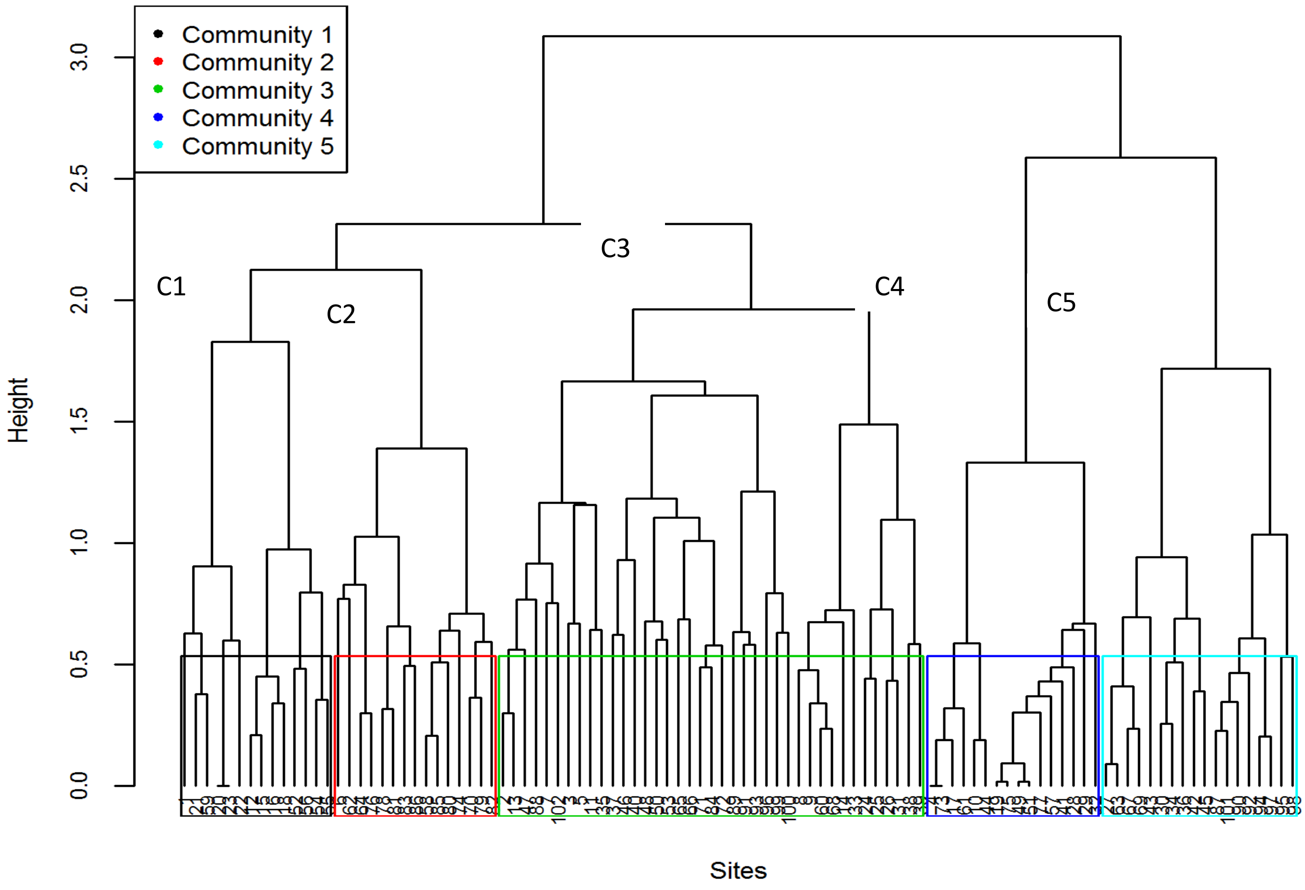

Figure 4: Dendrogram showing plant community types of Lake Abaya Wetland vegetation.

1. *Cyperus articulatus* community type: This community type was dominanted by *Cyperus articulatus. Cyperus laevigatus, Typha angustifolia, Cynodon aethiopicus, Aeschynomene elaphroxylon, Leersia hexandra* and *Eichhornia crassipes* were also important species with high mean cover values in the community type. This group included plant species that form wet meadow vegetation. Most of the plants in this community type were emergent macrophytes except few of them were floating plants such as *Nymphaea nouchali and Eichhornia crassipes*. Thus, in this community type, emergent plant species were more prominent than free floating, floating-leaf and submersed plants. This community type consists 13.7% of the stands most of which were from north and northwest of the lakeshore.

2. *Eichhornia crassipes-Pistia stratiotes* community type: Dominant species of this community type were *Eichhornia crassipes* and *Pistia stratiotes* (Figure 4.8). Species such as *Leptochloa fusca, Leersia hexandra, Cyperus articulatus* and *Aeschynomene elaphroxylon* also contributed high percentage of cover to this community type. This community type was dominated by aquatic and mainly hydrophytic or wetland dependent plant species that are tolerant to high water table. *Eichhornia crassipes, Nymphaea lotus*, *Pistia stratiotes*, *Wolffia arrhiza* and *Spirodela polyrrhiza* were free floating and floating-leaf plants in this community type. In this community type, free floating and floating-leaf plants were more prominent than emergent plants, but no submersed plants recorded. These stands were mainly distributed at the edge of the open water bodies.

***3. Cynodon aethiopicus* community type:** This transitional vegetation type was rich in species and dominanted by *C. aethiopicus and Leersia hexandra.* Tree species recorded from this community type were *Ficus sycomorus*, *F. ovata, F. sur,* and *Kigelia africana*. Free floating and floating-leaf plants in this community type include *Eichhornia crassipes, Nymphaea nouchali, Wolffia arrhiza* and *Pistia stratiotes.* This community type was composed of both wetland and upland species and represented species rich community. It had a higher proportion of upland species than the other communities. Thus, free floating and floating-leaf plant species were less prominent than emergent species. All types of floristic composition (forbs, grasses, trees, shrubs and sedges) were observed. This type was distinct from the other wetland communities and showed species richness and composition approaching that of upland vegetation. This community type consisted of 32.5% of the stands which were sampled from south east, southwest and south end of the lake.

4. *Typha angustifolia-Aeschynomene elaphroxylon* community type: This community type (Figure 4.9) was dominanted by *Typha angustifolia* and *Aeschynomene elaphroxylon. Leersia hexandra* was another important species contributing most to the composition of this community type. *Ficus sycomorus* and *Aeschynomene elaphroxylon* were tree species in this community type. This community type consisted of 15.7% of the stands which were from north, northwest, and west of the lake. All of the plants in this community type were emergent macrophytes except *Potamogeton pucillus,* a submerged aquatic plant. In this community type, free floating and floating-leaf plants were not recorded. Emergent plants were more prominent than free floating, floating-leaf and submersed plants.

***5. Aeschynomene elaphroxylon* community type:** This shruby marsh community type was dominanted by *Aeschynomene elaphroxylon* (Figure 4.10). *Nymphaea nouchali*, *Echinochloa pyramidalis, Leptochloa fusca, Eichhornia crassipes* and *Cyperus articulatus were* sub dominant species in this community type. This community type was represented by 17.6% of the stands sampled from north, northwest, west, and northeast of the lake. Free floating and floating-leaf plants recorded from this community type were (*Nymphaea nouchali, Pistia stratiotes, Spirodela polyrrhiza* and *Eichhornia crassipes*). Similar to community types two and four, this community type occurred on the edges of open and deeper standing water.

Table 1: Synoptic table of Lake Abaya Wetland vegetation showing mean cover values of species within clusters.

Species	Community types				
	C1	C2	C3	C4	C5
Acacia montigena	0.000	0.000	0.308	0.000	0.000
Acacia seyal	0.000	0.000	0.846	0.000	0.000
Acacia tortilis	0.000	0.000	0.128	0.000	0.000
Acalypha fruitcosa	0.000	0.000	0.436	0.000	0.000
Acalypha racemosa	0.000	0.000	1.359	0.000	0.000
Achyranthes aspera	0.000	0.000	0.795	0.000	0.000
Aeschynomene elaphroxylon	1.286	2.667	0.179	**4.750**	**7.111**
Ajuga leucantha	0.000	0.000	0.128	0.000	0.000

Aloe otallensis	0.000	0.000	0.308	0.000	0.000
Amaranthus dubius	0.000	0.000	0.282	0.000	0.000
Amaranthus spinosus	0.000	0.000	0.128	0.000	0.000
Phalaris arundinacea	0.000	0.467	0.000	0.000	0.000
Balanites aegyptiaca	0.000	0.467	0.589	0.000	0.000
Carissa spinarum	0.000	0.000	0.026	0.000	0.000
Cayratia ibuensis	0.000	0.000	0.069	0.000	0.000
Cissus quadrangularis	0.000	0.000	0.385	0.000	0.000
Clausena anisata	0.000	0.000	0.051	0.000	0.000
Commelina diffusa	0.000	0.000	0.103	0.000	0.000
Cordia africana	0.000	0.000	0.359	0.000	0.000
Croton macrostachyus	0.000	0.000	0.564	0.000	0.000
Cynodon aethiopicus	1.642	2.533	**3.564**	0.000	1.722
Cyperus alopecuroides	0.000	0.400	0.410	0.375	0.000
Cyperus articulatus	**4.714**	1.933	0.154	0.000	1.111
Cyperus digitatus	0.000	0.000	0.128	0.000	0.000
Cyperus distans	0.000	0.000	0.154	0.000	0.000
Cyperus grandibulbosus	0.429	0.000	0.000	0.000	0.000
Cyperus laevigatus	2.357	0.267	0.282	0.000	0.000
Cyperus latifolius	0.000	3.133	0.000	0.000	0.000
Cyperus papyrus	0.000	0.000	0.000	0.438	0.000
Cyperus rotundus	0.000	0.000	0.179	0.000	0.000
Cyperus subumbellatus	0.000	0.000	0.436	0.000	2.444
Echinochloa haploclada	0.500	0.000	0.000	0.000	0.000
Echinochloa pyramidalis	0.000	0.000	0.436	0.625	1.333
Eclipta prostrata	0.000	0.333	0.026	0.000	0.000
Eichhornia crassipes	0.923	**5.433**	0.154	0.000	1.222
Enneapogon desvauxii	0.357	0.000	0.385	0.000	0.000
Eragrostis japonica	0.000	0.000	0.154	0.375	0.000
Eriochloa fatmensis	0.000	0.000	0.000	0.250	0.000
Euphorbia tirucalli	0.000	0.000	0.154	0.000	0.000
Ficus ovata	0.000	0.000	0.538	0.000	0.000
Ficus sur	0.000	0.000	0.308	0.000	0.000
Ficus sycomorus	0.000	0.000	1.000	0.438	0.000
Geigeria alata	0.000	0.000	0.231	0.000	0.000
Gossypium barbadense	0.000	0.000	0.179	0.000	0.000
Grewia ferruginea	0.000	0.000	0.128	0.000	0.000
Grewia villosa	0.000	0.000	0.000	0.000	0.278
Hippocratea africana	0.000	0.000	0.154	0.000	0.000
Hypoestes forskaolii	0.000	0.000	0.231	0.000	0.000
Ipomoea aquatica	0.000	0.133	0.000	0.500	0.556
Ipomoea eriocarpa	0.000	0.000	0.077	0.563	0.167
Kigelia africana	0.000	0.000	0.179	0.000	0.000

Leersia hexandra	2.643	2.667	2.154	0.688	0.556
Leptadenia arborea	0.000	0.000	0.051	0.000	0.000
Leptochloa fusca	0.357	2.067	0.026	0.563	1.556
Leucas deflexa	0.000	0.000	0.179	0.000	0.000
Ludwigia stolonifera	0.000	0.000	0.103	0.000	0.000
Mangifera indica	0.000	0.000	0.051	0.000	0.000
Maytenus arbutifolia	0.000	0.333	0.154	0.000	0.000
Maytenus senegalensis	0.000	0.000	0.538	0.000	0.111
Melhania ovata	0.000	0.200	0.000	0.000	0.000
Nymphaea lotus	0.000	0.600	0.000	0.000	0.000
Nymphaea nouchali	0.429	0.000	0.128	0.000	1.556
Phragmates karka	0.000	0.000	0.000	0.313	0.000
Pistia stratiotes	0.000	**4.533**	0.128	0.000	0.389
Plecthrantus barbatus	0.000	0.000	0.128	0.000	0.000
Pluchea ovalis	0.000	0.000	0.872	0.000	0.000
Potamogeton pucillus	0.000	0.200	0.000	0.125	0.000
Pulcaria schimperi	0.000	0.000	0.077	0.000	0.000
Senna didymobotrya	0.000	0.000	0.359	0.000	0.333
Senna septemtrionalis	0.000	0.000	0.000	0.000	0.278
Sesbania sesban	0.000	0.067	0.179	0.000	0.000
Solanum incanum	0.000	0.000	0.949	0.000	0.000
Sorghum verticilliflorum	0.357	0.333	0.205	0.813	0.000
Spirodela polyrrhiza	0.000	0.000	0.026	0.000	0.222
Trichilia emetica	0.000	0.000	0.231	0.000	0.000
Typha angustifolia	2.471	0.400	0.308	**7.875**	0.000
Withania somnifera	0.000	0.000	0.077	0.000	0.000
Wolffia arrhiza	0.000	0.533	0.179	0.000	0.000
Xanthium strumarium	0.000	0.000	0.615	0.063	0.333

Table 2: Summary of wetland plant community types from Lake Abaya vegetation data.

**Agglomerative hierarchical relationships**	**Plant community types**				
	**1**	**2**	**3**	**4**	**5**
**Dominant species**	*Cyperus articulatus*	*E. crassipes*<br>*P. stratiotes*	*Cynodon aethiopicus*	*T. angustifolia*<br>*A.elaphroxylon*	*Aeschynomene elaphroxylon*
**Mean cover values**	4.71 and 3.36	5.43 and 4.53	3.56	7.88 and 4.75	7.11
**Sub-dominant species**	*T. angustifolia, C. aethiopicus, A.elaphroxylon* and *L. hexandra*	*L. fusca,*<br>*L. hexandra,*<br>*C. articulatus, C. aethiopicus,* and *A.elaphroxylon*	*Leersia hexandra, F. sycomorus* and *A. fruitcosa*	*Leersia hexandra*	*C. aethiopicus, N. nouchalii, E. pyramidalis,*<br>*L. fusca,*<br>*E. crassipes, C. articulatus*

**Shannon's diversity x(H)**	2.10	2.56	**3.64**	1.87	2.31
**Shannon's evenness (J)**	0.84	0.82	**0.87**	0.67	0.80
**Richness (S)**	12	23	**67**	16	18
**No. quadrats**	14	15	**39**	16	18

3.1.2. Diversity of Plant Community Types

The Shannon-Wiener Diversity computed for five different plant communities (Table 2) showed that community type three (Cynodon aethiopicus) which was found in relatively dry terrestrial landscape and exposed for disturbance was the most diverse and has the highest species richness and eveness. Community type four (Typha angustifolia-Aeschynomene elaphroxylon) which occurred in deeper standing water around the open water zones and experiencing prolonged inundation had the least diversity index and had the least even distribution of species. Community type one (Cyperus articulatus) was the least in species richness and occurred in deeper water next to community four.

3.2. Environmental Gradients Influencing Wetland Plant Communities

3.2.1. Multivariate Non-metric Multidimensional Scaling (NMDS)

Multivariate Non-metric Multidimensional Scaling (NMS) (McCune and Grace 2002) resulted in a 2-dimensional solution with a final stress of 0.255 (Figure 5). The first axis was most correlated with water depth (r^2 =0.248, p=0.001), Hydrogeomorphology (r^2 =0.167, p=0.003) and slope (r^2 =0.334, p=0.001) (Table 3). Community type five (*Aeschynomene elaphroxylon*) was mostly correlated with drainage (r^2 =0.067, p=0.039) where as community type three was highly correlated with slope r^2 =0.334, p=0.001)(Table 3). *Typha angustifolia-Aeschynomene elaphroxylon* (community four) which mainly consisted of wetland dependent species was correlated with higher water depth (r^2 =0.248, p=0.001)(Table 3).

Table 3. Pearson (r^2) correlations of Nonmetric Multidimensional Scaling ordination axes with environmental variables of Lake Abaya vegetation. Significance codes: 0 '***' 0.001 '**' 0.01 '*' 0.05 '.' 0.1 ' ' 1, P values based on 999 permutations

**Vectors**	**NMDS1**	**NMDS2**	**r^2**	**Pr(>r)**
Depth	-0.793	-0.609	0.248	0.001***
Drainage	-0.942	0.335	0.067	0.039
Hydrogeo	0.906	0.423	0.167	0.001***
Slope	0.968	0.250	0.334	0.001***

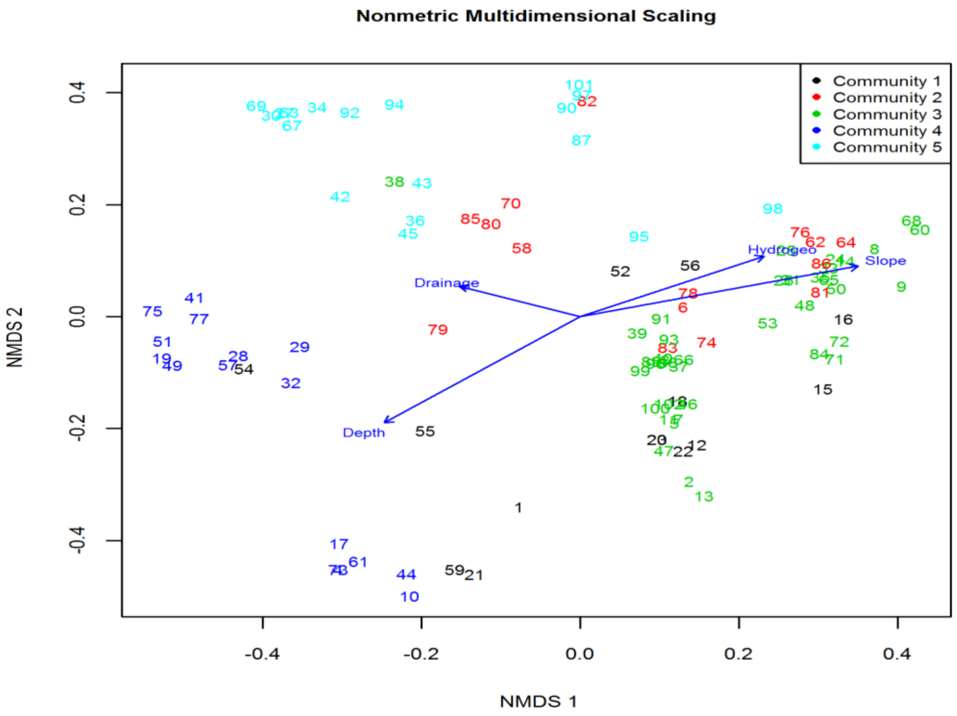

Figure 5. Non-metric Multidimensional Scaling (NMDS) diagram of plots of Lake Abaya vegetation grouped by community type (communities 1 to 5). Vectors represent environmental variables: depth, hydrogeomorphology, drainage and slope.

3.2.2. Canonical Correspondence Analysis (CCA)

Species and plots ordination in the space defined by the first two CCA axes are shown in Figures 6a and 6b. CCA ordination illustrates the relationship of the four environmental variables (water depth, drainage, slope and hydrogeomorphology) to five community types and to species distribution pattern. The first two axes together accounted for 63% (Table 4.8) of the variation explained. The proportion of variances explained by these axes was 36% and 27%, respectively (Table 4).

Results from ANOVA test showed that water depth, slope, drainage and hydrogeomorphology were significantly related to the floristic composition of the plant community types. The first axis extracted by the analysis was closely related to the drainage and slope, as indicated by the biplot scores of -0.43 and -0.78 (Table 4). The second axis extracted by the analysis was more closely related to water depth and hydrogeomorphology, as indicated by the biplot scores of -0.71 and 0.54, respectively (Table 4). The second axis was associated negatively with water depth and positively with hydrogeomorphology.

Table 4. Results of CCA analysis of Lake Abaya wetland vegetation data. Canonical coefficients from the best-fit multiple regression models (ANOVA test), biplot scores for the constraining variables, eigenvalues and proportion of variances explained by the first two axes were indicated. Factor values that differed significantly between the groups according to an ANOVA are shown in bold.

**Variables**	**CCA1**	**CCA2**	**SSqs**	**MSqs**	**F**	**R2**	**Pr(>F)**
Slope	**-0.78**	0.60	1.32	1.32	3.78	0.03	0.01**
Depth	-0.38	**-0.71**	2.13	2.13	6.10	0.05	0.01**

Hydrogeomorphlogy	0.41	**0.54**	0.71	0.71	2.02	0.02	0.05*
Drainage	**-0.43**	-0.31	0.97	0.97	2.78	0.02	0.01**
Eigenvalues	0.41	0.31					
% variance explained	0.36	0.27					
Cumulative % variance	0.36	0.63					
Species enviroment correlations	0.85	0.74					

(* = 0.01 < p < 0.05, ** = p < 0.01 and *** = p < 0.001).

Ordination diagram of CCA (Figure 6a) for plots and environmental factors showed that *Eichhornia crassipes-Pistia stratiotes* (community two) and *Typha angustifolia-Aeschynomene elaphroxylon* (community four) were found in areas with high water level and poor drainage. Therefore, distribution of these community types was significantly affected by hydrologic factors such as water depth, drainage and hydrogeomorphology. These community types were consisted of wetland dependent plant species that are tolerant to high water table which most commonly occur in waterlogged areas characterized by presence of standing or permanently flowing water for most of the year. This wetland dependent community type also occurred in permanently flooded areas as well as in and around the edge of open water bodies.

Alternatively, *Cynodon aethiopicus* (community type 3) with large number of plots (in the upper part of the ordination space (Figure 6b) was found in excessively drained areas with high disturbance and low water level. These groups also commonly occurred in areas characterized by low lying dry terrestrial landscape with well drained soils. Water depth and hydrogeomorphology were strong factors differentiating these groups from other community types. Slope was a strong factor differentiating community type five (*Aeschynomene elaphroxylon*) from others. It was relatively intact and pristine or semi-natural vegetation found in areas occasionally used for grazing activities.

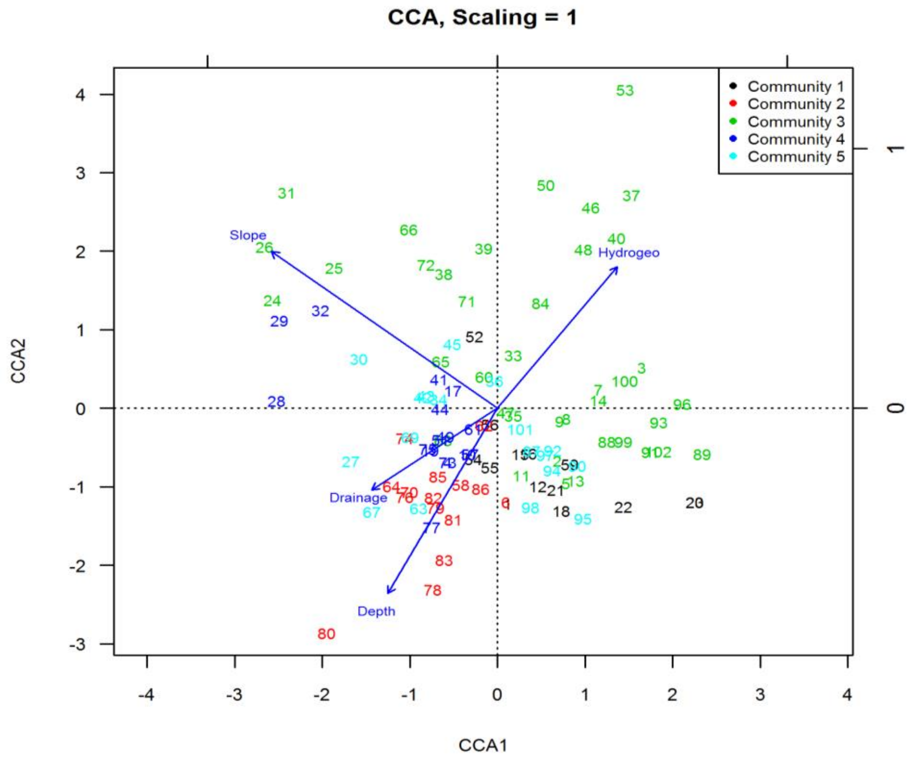

(a)

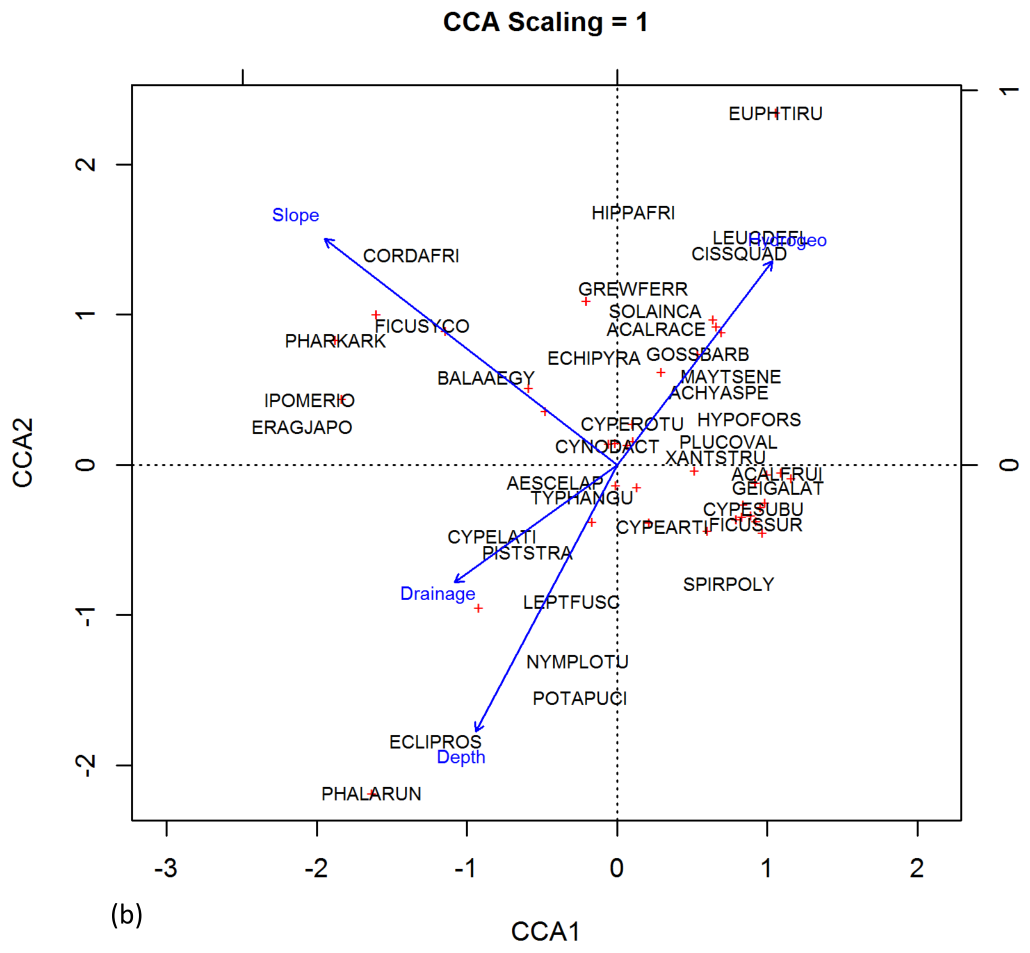

Figure 6. Canonical correspondence analysis (CCA) of wetlands of Lake Abaya vegetation. (a): shows the relationships between the plant community types and environmental variables, (b): shows the relationships between plant species distribution and environmental variables. Only higher priority species with high variances are visible and all other less dominant species are indicated by plus sign (+). Species were abbreviated by combining the first four letters from generic names and specific epithets. Environmental attributes represented by the vectors included water depth, slope, drainage and hydrogeomorphology. The vectors representing environmental gradients point in the direction of the most rapid change in each variable.

Analysis of variance showed that there was a strong relationship between species and environmental factors such as water depth ($p<0.01$), drainage ($p<0.01$), hydrogeomorphology ($p<0.05$) and slope ($p<0.01$). The species and environment correlations with the first axis was 0.85 and the second axis was 0.74 (Table 4). Canonical correspondence analysis (CCA) (Figure 6b) for the species and explanatory variables revealed essentially the same pattern as ordination of plots and explanatory variables.

The analysis of variance also showed that there was a marked relationship between the environment and the scores of many species. The plant species *Pharagmatis karka*, *Cordia africana*, *Ficus sycomorus* and *Eragrostis japonica* had strong relationships with the first axis (more closely related to slope). *Euphorbia tirucalli*, *Hippocratea africana* and *Leucas deflexa* were more closely related to areas with decreasing water depth and drainage), whereas *Phalaris arundinacea*, *Eichhornia crassipes*, *Pistia stratiotes*, *Nymphaea lotus* and *Potamogeton pucillus* had significant relashinship with increasing water depth and drainage.

4. Discussion

The ordination diagram displayed a measure of overlap among vegetation types and also indicated various groups (plant community types) that uniquely occupy ordination space. One of the insights obtained from the ordination process was that the dominant graminoids (with several exceptions) were rather broadly distributed and overlapping in ordination space. Furthermore, the study on the wetlands resulted in plant communities of emergent species, aggregating free floating, floating leaf and submerged plants as minor members of associations dominated by the more obvious emergent species and did not indicate the differences that may exist between emergent and submerged plants as distinct components of wetland communities. This could be due to shared affinities for environmental conditions.

Disturbance in the form of burning, land use types, drainage, pollution, eutrophication or overgrazing can reduce plant species diversity by eliminating disturbance sensitive species (Connell 1978). Drainage for cultivation can affect ground water table and in turn the organic matter content of the soil. Species losses occur as a result of habitat loss and fragmentation of ecosystem because of direct human activities, including trampling and collecting. Trampling associated to fishing could be responsible for the lower number of plant species (diversities) encountered in the wetland.

Sileshi Bekele (2001) pointed out that Lake Abaya and Lake Chamo wetlands which receive agricultural inputs and waste water predominantly from human settlements in the catchment and surrounding highlands were found to experience eutrophication with higher levels of nutrients. Eutrophication is a gradual increase in the concentration of phosphorus, nitrogen and other plant nutrients in aquatic systems, which results in the system becoming more productive. Wetlands fed by surface water from agricultural and urbanized watersheds tend to have invasive species (Galatowitsch et al. 1999). Manure transported from the surrounding areas under cultivation has an additional effect that contributed to eutrophication. Therefore, eutrophication may have caused the Lake to be more productive through nutrient enrichment and hence causing the wetlands to be invaded by invasive species and agricultural weeds. Similar situation has been reported in other wetlands with high eutrophication level in Africa by Gichuki et al. (2001), Abila et al. (2008).

The presence of introduced or invasive species and their high frequency around the wetlands might indicate a strong anthropogenic disturbance effect of land use on these wetlands. Human related disturbances are well known to facilitate successful establishment of non-native (including invasive species) and weedy plant species in many vegetation types (Hobbs 1991; Hobbs and Huenneke 1992). It is therefore, possible that an expansion of cultivated land and associated eutrophication process and a change in grazing regime, due to increase in the number of cattle in the wetlands had contributed to the current spatial heterogeneity in species composition of weedy and invasive plant species. Most of the invasive and weedy plant species take advantage of the changes in site conditions, especially increase in nutrients following a significant disturbance (Milbau and Andi 2004). Large herbivores influence species composition by removing herbage, trampling and through dung and urine depositions (Hobbs 1991). Dung deposition modifies site conditions by supplying nutrients and facilitates seed dispersal (Hobbs 1991). Rea and

Storrs (1999) and DiTomaso (2000), reported that increase in cattle grazing effects such as trampling and nutrient inputs may accelerate the spread of invasive which consequently changes plant species composition and functional structure.

5. Conclusion and Recommendations

The study has resulted in the documentation of 92 plant species representing 66 genera and 34 families. Most of the plants in the wetlands under investigation were emergent macrophytes except a few of them that were free-floating, floating-leaf and submerged species. The presence of weedy, introduced or invasive species and plant community types dominated by these species indicate a relatively more anthropogenic disturbance effect of land use on wetlands of Lake Abaya.

The distribution and composition of plant species and vegetation assembly at study site were influenced significantly by a combination of different environmental factors. In the wetland, the dominant grasses and sedges were widely distributed resulting in an overlap among plant community types. Furthermore, the study on the wetlands resulted in plant communities of mainly emergent species, aggregating free floating, floating-leaf and submerged plants as minor members of associations dominated by the more obvious emergent species and did not indicate the differences that may exist between emergent and submerged plants as distinct components of wetland communities. Thus, the presence of non-wetland shrub and forb species and their high frequency in the wetlands indicated that the wetland was experiencing disturbances from both natural environment and anthropogenic factors.

Results of the study showed that there were differences in the spatial and temporal patterns of plant species composition and diversity due to variations in environmental gradients (hydrologic factors in particular) and anthropogenic factors. Therefore, this should be considered in future management and protection under the circumstance of climate change and human activities. Overall, results support the prediction that different environmental variables had varying influence on the overall plant species composition, diversity and distribution.

Long term monitoring of the wetland plant communities is recommended as the environment changes with increased human activities that are likely to increase the abundance of invasive species. The number and cattle movements should be controlled in order to maintain the integrity of the wetland.

Protective measures should be developed to safeguard the study areas from destructive anthropogenic impacts; otherwise the damage can become irreversible. The effective and continuous protection measures should be identified and local community should be informed about the importance of the conservation of these fragile ecosystems. Wetland managers and decisions makers at all levels should consider this baseline data on the species composition and vegetation ecology to guide management decisions and to detect changes over time and space as a result of management and impacts of anthropogenic alteration.

Further study of soil nutrients and water chemistry data is recommended to better understand the compositional variation found between and among the wetlands. In addition, an assessment of the vegetation in terms of its variability and reaction to various practices such as burning and grazing should be done.

Statements and Declarations

Funding: The authors declare that no funds or grants were received during the preparation of this manuscript.

Competing Interest: The author declare that there is no competing interests that could have appeared to influence the work reported in this paper. The content of the manuscript has not been published, or submitted for publication elsewhere.

Author Contributions: The original study conception and design, field survey plan, material preparation, data collection, analysis and the first draft and final write up of the paper were performed by Dikaso Unbushe.

Acknowledgement

The author would like to acknowledge the National Meteorological Agency for providing free temperature and rainfall data. The staff members of the National Herbarium, Ethiopia, are also greatly acknowledged. The authors declare that there is no conflict of interest. Addis Ababa University supported material required for this study.

References

Abila, R., Salzburger, W., Ndonga, M.F., Owiti, D.O., Barluenga, M. and Meyer, A. (2008). The role of the Yala swamp lakes in the conservation of Lake Victoria region haplochromine cichlids: Evidence from genetic and trophic ecology studies. *Lakes Reservoirs Res Manage* **13**: 95-104.

Afework Hailu, Dixon, A.B. and Wood, A.P. (2000). Nature, extent and trends in wetland drainage and use in Illubabor Zone, southwest Ethiopia. Ethiopian Wetlands Research Programme (EWRP) and the University of Huddersfield. Huddersfield.

Barbier, E.B., Acreman, M.C. and Knower, D. (1996). Economic Valuation of Wetlands. A guide for policy makers and planers. Ramsar Convention Bureau, Gland, Switzerland. PP. 127.

Bayafers Tamene, Tamrat Bekele and Ensermu Kelbessa (2000). An Ethnobotanical study of the Semi-wetland Vegetation of Cheffa. NAPRECA, Addis Ababa University. Addis Ababa Ethiopia

Connell, J.H. (1978). Diversity in tropical rain forests and coral reefs. *Science* **199**: 1302-1310.

Costanza, R., Arge, R., Groot, R., Faber, S. and Grasso, M. (1997). The Values of the World's Ecosystem Services and Natural Capital. *Nature* **387**: 253-260.

DiTomaso, J.M. (2000). Invasive weeds in rangelands: species, impacts, and management. *Weed Sci.* **48**:255–265.

EMA /Ethiopian Mapping Agency/ (1988). A National Atlas of Ethiopia. Ethiopian Mapping Agency, Addis Ababa.

Enserink, M. (1999). Predicting invasions: biological invaders sweep in. *Science* **285:**1834–1836.

EPA (2003). State of the Environment Report for Ethiopia. Environmental Protection Authority (EPA), Addis Ababa, Ethiopia.

Finlayson, M. and Moser, M. (1991). Wetlands. International Waterfowl and Wetland Research Bureau. Facts on File Ltd. Oxford, UK. PP. 224.

Galatowitsch, S. M., Anderson, N. O., and Ascher, P. D. (1999). Invasiveness in wetland plants in temperate North America. *Wetlands* **19**: 733–755.

Gichuki, J.F., Dahdouh, G.F., Mugo, J., Rabuor, C.O., Triest, L., and Dehairs, F. (2001). Species Inventory and the Local uses of the Plants and the Fisheries of the Lower Sondu Miriu Wetland of Swamp Victoria, Kenya. *Hydrobiologia* **458**: 99-106.

Hill, M.O. (1979). DECORANA–FORTRAN program for detrended correspondence analysis and reciprocal averaging. Ecology and Systematics, Cornell University, New York.

Hobbs, R. (1991). Disturbance as a precursor to weed invasion in native vegetation. *Plant Prot.* **6**:99–104.

Hobbs, R.J. and Huenneke, L.F. (1992). Disturbance, diversity, and invasion: implications for conservation. *Conserv Biol*. **6**:324–337.

Kent, M. and Coker, P. (1992). Vegetation Description and Analysis. A Practical Approach. Belhaven Press, London.

Lean, G., Hinrichsen, D. and Markham, A. (1990). Atlas of the Environment. Benson Marketing Ltd., London, UK. PP. 192.

Ludwig, J. A., and Reynolds, J. F. (1988). Statistical Ecology. John Wiley and Sons.

Maltby, E.C. (1986). Waterlogged wealth: why waste the world's wet places? Earthscan, London, UK. PP. 200.

McCune, B. and Mefford, M.J. (1997). Multivariate Analysis of Ecological Data. Version 3.09. MjM Software, Gleneden Beach, Oregon, USA.

McCune, B. and Grace, J.B. (2002). Analysis of ecological communities. MjM Software Design, Gleneden Beach, Oregon.

Milbau, A. and Andi, N. (2004). The role of species traits (invasiveness) and ecosystem characteristics (invasibility) in grassland invasions: a framework. *Weed Technol.* **18**:1301–1304.

Mitiku Tikssa, Tamrat Bekele and Ensermu Kelbessa (2010). Plant community distribution and variation along the Awash river corridor in the main Ethiopian rift. *Afr. J. Ecol*. **48**(1):21-28.

Mitsch, W.J. and J.G. Gosselink (1993). Hydrology of wetlands. In Wetlands. 2nd ed. Mitsch and Gosselink (eds.)Van Nostrand Reinhold, New York.

Mitsch, W.J. and Gosselink, J.G. (2002). Wetlands. 3rd ed, John Wiley and Sons, Inc. U.S.A.

Mueller-Dombois, D. and Ellenberg, H. (1974). Aims and Methods of Vegetation Ecology. John Wiley and Sons, Inc. New York.

OECD (1996). Guidelines for Aid Agencies for Improved Conservation and Sustainable Use of Tropical and Sub-Tropical Wetlands, France, PP 67.

R Development Core Team (2009). R: A language and environment for statistical computing. R Foundation for Statistical Computing, Vienna, Austria. http://www.R project.org.

Rea, N., and Storrs, M.J. (1999). Weed invasions in wetlands of Australia's Top End: reasons and solutions. *Wetland Ecol. Management* **7**:47–62.

Schuyt, K. (2004). The Economic values of the world's Wetlands. WWF International, Gland, Switzerland.

Seleshi Bekele (2001). Investigation of Water Resources Aimed at Multi purpose Development with Limited Data Situation: The Case of Abaya-Chamo Basin, Ethiopia, Ph.D. Thesis, Dresden University of Technology, Germany, March 2001.

Shannon, C.E. and Wiener, W. (1949). The mathematical theory of Communication. University of Illonois Press.

ter Braak, C.J.F. (1987). Ordination. **In**: Data analysis in community and landscape ecology (Jongman, R.H.G., Ter Braak, C.J.F. & Tongeren, O.F.R., eds). Pudoc, Wageningen.

ter Braak, C.J.F. (1988). CANOCO–FORTRAN program for canonical community ordination by canonical correspondence analysis, principal components analysis and redundancy analysis. Agricultural Mathematics Group, Wageningen.

ter Braak, C.J.F. (1990). Update notes: CANOCO version 3.10. Agricultural Mathematics Group, Wageningen.

ter Braak, C.J.F. (1995). Canonical community ordination. Part II: The correspondence analysis family.

ter Braak, C.J.F. and Smilauer, P. (2002). CANOCO Reference Manual and CanoDraw User's Guide–Software for canonical community ordination. Microcomputer Power, Ithaca, New York.

Turner, R.K., van den Bergh, J.C., Söderqvist, T., Barendregt, A., van der Straaten, J., Maltby, E. and van Ierland, E.C. (2000). Special issue: The values of wetlands: Landscape and institutional perspectives. Ecological-economic analysis of wetlands: Scientific integration for management and policy. Ecological Economics **35**: 7-23.

Van der Maarel, E. (1979). Multivariate Methods in Phytosociology, with reference to the Netherlands. The study of vegetation. Junk, The Hague.

Wetzel, R.G. (1975). Limnology. R.G. Wetzel (ed.) Saunders College Pub., Philadelphia,PA.

Whittaker, R.H. (1975). Community and ecology. 2nd ed. Macmillan Publishing Co. Inc. New York.
