# Supplementary material for: Wetland vegetation composition and ecology of Lake Abaya in southern Ethiopia
Source: PLoS One. 2024 Apr 10;19(4):e0301795. doi: 10.1371/journal.pone.0301795 (PMC11006174; doi:10.1371/journal.pone.0301795)
Supplement: Attachment — Submitted filename: To the Editor and the author.pdf [file pone.0301795.s003.pdf]

To the Editor and the author

Introduction: The often overlooked functions of wetlands, such as their role in purification and carbon sequestration, can diminish the perceived significance of these ecosystems and consequently reduce the motivation to conserve them, particularly within the perspective of policy makers.

Study Area: The graphical representation of the study area depicted in Figure 1 raises concerns due to its poor quality. The aspect ratio of latitude and longitude coordinates is not accurately represented, necessitating a revision of the map to ensure an appropriate scale and resolution. Moreover, the climadiagram's horizontal axis exhibits an exaggeration that may distort its true representation.

Data Collection: The explanation provided for the methods employed in data collection lacks comprehensive detail. A more in-depth description could draw insights from the approach employed in measuring drainage and slope, aiding in establishing a clearer understanding of the study's data gathering procedures.

Data Analysis: The utilization of an outdated version of R during the data analysis, when compared to the more recent version 4.3.1, raises concerns. Employing an outdated version has the potential to influence the outcomes of data analysis, as is evident in the substandard quality of the dendrogram presented in Figure 4. Enhancements such as the incorporation of colored leaves (branches) and labels associated with the required number of clusters can considerably improve the dendrogram's effectiveness. While the dendrogram demonstrates differences in clusters, the rationale behind selecting cluster identification heights spanning the dendrogram's various levels is provided, even when the dissimilarity between the clusters is significant. A decision to recognize just four distinct clusters arises when the cutoff height falls between 2 and 2.5.

The application of NMDS (Non-Metric Multidimensional Scaling) facilitates the correlation of environmental factors with the clusters that have been identified. In cases where NMDS has already produced satisfactory outcomes, the relevance of employing CCA (Canonical Correspondence Analysis) in establishing the relationship between sample plots and environmental factors requires a compelling justification.

What is the significance of the red cross symbols within Figure 6, representing the Canonical Correspondence Analysis (CCA) involving species and environmental factors? Can these symbols be eliminated if needed?
