# Supplementary material for: Wetland vegetation composition and ecology of Lake Abaya in southern Ethiopia
Source: PLoS One. 2024 Apr 10;19(4):e0301795. doi: 10.1371/journal.pone.0301795 (PMC11006174; doi:10.1371/journal.pone.0301795)
Supplement: Attachment — Submitted filename: Response to Reviewers (3).docx [file pone.0301795.s006.docx]

**Response to Additional Editor Comments**

- Response to reviewers comments submitted as an attachment file were previously uploaded as a rebuttal letter that responds to each point raised by the academic editor and reviewer(s). This letter was uploaded as a separate file labeled 'Response to Reviewers' as shown below. However, I have received similar file as attachment for second time.
- I have uploaded the figure files to the Preflight Analysis and Conversion Engine (PACE) digital diagnostic tool, so as to ensure that figures meet PLOS requirements.
- I have reviewed the reference list to ensure that it is complete and correct.
- I have cross-checked the papers cited in the manuscript against The Retraction Watch Database online and found no retracted article have been cited.

**Response to Reviewers**

- Some typographical or grammatical errors has been corrected at revision to make the article clear, correct, and unambiguous.

Reviewer #1:

1. The manuscript is generally well written with adequate statistical analysis. However, the discussion section is thin with some results not discussed at all.

- To make the discussion section strong, explanation on the relationship of some important factors with plant community types is provided. Discussion of results on plant diversity and distribution is included and some discussions unrelated to results has been avoided to match the amount of results presented.

2. The author needs to state how sampling effort was measured.

- The total number of plots, and the total area sampled, plot sizes and their intervals (distance) between plots used in different vegetation types were stated clearly and the references cited.

3. The citation of references is good but majority are quite old considering the dynamism in wetlands loss/degradation.

- Some recent references on the status of wetlands cited.

4. The recommendations and conclusions do not clearly originate from the results perhaps due to the thin discussion. In fact some appear to be from general knowledge.

- The conclusion and recommendation section is updated to match the results and some general facts removed.

5. Most of the comments are included in the manuscript as comments

- The manuscript is edited as per the comments included

Reviewer #2: To the Editor and the author

Introduction: The often overlooked functions of wetlands, such as their role in purification and carbon sequestration………

- The role of wetlands in water purification and carbon sequestration included in the introduction section.

Study Area: The graphical representation of the study area depicted raises concerns due to its poor quality. The aspect ratio of latitude and longitude coordinates is not accurately represented, necessitating a revision of the map to ensure an appropriate scale and resolution.

- The map (Figure 1) is revised or drawn again to ensure an appropriate scale and resolution and to accurately represent aspect ratio so as to enhance its quality. The soil type of the study area and some water parameters were briefly described

Data Collection: The explanation provided for the methods employed in data collection lacks comprehensive detail……..

- To provide detail explanation for the methods employed in data collection, further elaborations made. The total number of plots, and the total area sampled, plot sizes and their intervals (distance) between plots used in different vegetation types were stated clearly and the references cited. The table consisting of environmental variables recorded and rated from the study sites is included in the manuscript.

Data Analysis: The utilization of an outdated version of R during the data analysis, when compared to the more recent version 4.3.1, raises concerns……..

- The data from which dendrogram was derived is also checked with the more recent version 4.3.1 and similar graphics is produced. The cutoff height was chosen just above 2 based on the researcher’s ecological evaluation or observation in the field, cluster analysis output and the resulting synoptic table, and hence five community types were selected. As mentioned in most literature, researchers can choose to develop a cluster analysis when their main goal is to sort and allocate observations to groups and, from then on, to analyze what the ideal number of clusters formed is based on field observation and results from software analysis.

The application of NMDS (Non-Metric Multidimensional Scaling) facilitates the correlation of environmental factors with the clusters………

- Although both methods facilitate the correlation of environmental factors with the clusters or sample plots, the idea is to depict the most accurate representation of relationships of observations/measurements/data points between both categories. CCA sometimes performs well with skewed species distributions, with quantitative noise in species abundance data.

What is the significance of the red cross symbols within Figure 6, representing the Canonical Correspondence Analysis (CCA) involving species and environmental factors? Can these symbols be eliminated if needed?

- Species were abbreviated by combining the first four letters from generic names and specific epithets and only higher priority species with high variances are visible and all other less dominant species are indicated by red cross or plus sign (+).

**Response to Academic editors**

**Journal Requirements:**

1. Please ensure that your manuscript meets PLOS ONE's style requirements, including those for file naming

- The manuscript is carefully formatted and edited following PLOS ONE style requirements and templates.

2. During our internal evaluation of the manuscript, we found significant text overlap between your submission and previous work……..

- I confirm that this manuscript is part of my PhD study work. It has not been published elsewhere and is not under consideration by another journal. Overlapping and duplicating texts in the manuscripts have been rephrased and sources are cited.

3. Thank you for stating the following financial disclosure: …….

- I confirm that this manuscript is part of my PhD study work. The author received no specific funding for this work.The amended statement is also included within the cover letter.

4.In your Data Availability statement, you have not specified where the…….

- Data Availability statement: Study data is available based up on request. The minimal underlying data is uploaded as supporting information file.

5.We note that [Figure 1] in your submission contain [map/satellite] images which may be copyrighted………

- Figure 1 is replaced with new map. I confirm that it is taken from my PhD work.
- The source of shape file is central statistical agency of Ethiopia, base maps is from Geo-spatial information institution of Ethiopia and Images are from US GEOLOGICAL SURVEY/European space agency/Geo-spatial information institution of Ethiopia
